# Blockchain Based Transaction System with Fungible and Non-Fungible Tokens for a Community-Based Energy Infrastructure

**DOI:** 10.3390/s21113822

**Published:** 2021-05-31

**Authors:** Nikita Karandikar, Antorweep Chakravorty, Chunming Rong

**Affiliations:** Department of Electrical Engineering and Computer Science, University of Stavanger, 4021 Stavanger, Norway; antorweep.chakravorty@uis.no (A.C.); chunming.rong@uis.no (C.R.)

**Keywords:** blockchain, prosumer, fungible, NFT, peak shaving, smart grids, trading, demand response

## Abstract

Renewable energy microgeneration is rising leading to creation of prosumer communities making it possible to extract value from surplus energy and usage flexibility. Such a peer-to-peer energy trading community requires a decentralized, immutable and access-controlled transaction system for tokenized energy assets. In this study we present a unified blockchain-based system for energy asset transactions among prosumers, electric vehicles, power companies and storage providers. Two versions of the system were implemented on Hyperledger Fabric. Assets encapsulating an identifier or unique information along with value are modelled as non-fungible tokens (NFT), while those representing value only are modelled as fungible tokens (FT). We developed the associated algorithms for token lifecycle management, analyzed their complexities and encoded them in smart contracts for performance testing. The results show that performance of both implementations are comparable for most major operations. Further, we presented a detailed comparison of FT and NFT implementations based on use-case, design, performance, advantages and disadvantages. Our implementation achieved a throughput of 448.3 transactions per second for the slowest operation (transfer) with a reasonably low infrastructure.

## 1. Introduction

Renewable energy, especially solar energy is being increasingly integrated into the energy grid as photo voltaic installations continue to mushroom in residential contexts. This rise in adoption is fuelled partly by financial incentives like government programs and monetary benefits of local energy production [1] and in part by rising environmental awareness [2]. A new category of energy users called the prosumers [3] has been created, who generate a portion of the energy they consume through their local microgeneration devices. Several prosumers, when collocated give rise to prosumer communities or microgrids [4] and can create a local market for sale and purchase of surplus renewable energy. This creates a scenario for peer to peer energy transactions. Pilot studies in peer to peer prosumer trading [5], have been conducted with different business models [6] and have shown its importance in facilitating local power and energy balance [7]. Prosumer communities can also choose to store surplus energy for later use by exploring energy storage at the community level. Energy Storage as a service [8] is a burgeoning new business that takes over the logistics of setting up and maintaining a large scale storage facility and offers storage credits to the users for purchase. Such an arrangement can offer significant economic benefits [9]. Community level energy storage can facilitate energy transactions as energy can be supplied from the seller to the buyer via this storage, thus reducing the need for wired connections between all the members of the community. In addition, surplus stored energy can be used for powering Electric Vehicle (EV) charging stations, thus offering more opportunities for monetizing the surplus energy. EV batteries, when not in use can be rented out to the community level storage provider to add to the storage capacity. Mahmud et al. [10] proposed a system for using community level storage for charging EVs and presented a decision tree based algorithm for peak load reduction through coordinated management of EV, photovoltaics and community level storage.

Peak demand periods [11] present a challenge to the grid operator as they may require them to over provision grid capacity in order to maintain grid stability, thus increasing the marginal cost of electricity. Peak shaving strategies such as demand side response [12] are thus of particular importance to grid operators. An incentive based, direct to consumer demand response mechanism can be considered, allowing the power company to offer reward tokens to their customers in exchange for energy flexibility. As the prosumers are located in close proximity to each other, considering them as a prosumer community will allow the grid operator or power company to consider the required energy flexibility for the community as a whole [13]. In addition, the community structure can be used to increase interest and engagement by offering game based energy flexibility tasks [14].

Prosumers, EV owners, Power Companies and Storage Providers, by virtue of their relationships are stakeholders in a business network for transacting energy assets such as energy units, storage credits and reward tokens. An integrated solution is required to on-board the stakeholders and encapsulate the business network and relationships. As several small scale producers and consumers will constitute the network, a decentralized system is necessary in order to prevent the management from being concentrated in the hands of a single central entity. Moreover, all stakeholders must agree on the business logic and the transactions of energy assets must be immutable, transparent and verifiable to all. Provenance tracing of assets should also be enabled in order to make the system more trustworthy.

Blockchain [15] is a distributed shared ledger that fulfils these requirements [16,17]. Due to its characteristics of decentralization and immutability it prevents any member from unilaterally making decisions on the network [18,19]. Members are required to seek consensus before adding any transactions to the ledger and transactions are transparent to all members. Transaction history of assets on the blockchain can be readily traced for establishing provenance.

Blockchain implementations can be broadly categorized as permissioned or permissionless. Blockchain platforms such as Bitcoin [15] and Ethereum [20] are permissionless and allow anyone to join the network and perform transactions. Due to the anonymity inherent in these systems, computationally expensive consensus mechanisms such as Proof of Work are used due to the lack of any trust between transacting parties. Permissioned blockchain platforms such as Hyperledger Fabric [21] only permit authenticated parties to join the network and can have defined access permissions to dictate the privileges of each network member. As transactions are traceable to the invoking member, dependence on resource intensive consensus mechanisms is eliminated, thus reducing the operating cost.

Authentication of clients also helps the network satisfy know your customer and anti money laundering requirements [22] imposed by many countries. Moreover, Hyperledger Fabric supports the encoding of business logic into smart contracts using popular general purpose programming languages such as Golang [23] to create and modify assets or tokens on the network where a token is defined as anything of value. The algorithms defining the business logic encoded as smart contracts in this work have been presented in Algorithms 1–11.

Hyperledger Fabric’s modular architecture allows the operator to tailor implementation of trust models, transaction format and consensus mechanisms to the use case. Gur et al. [24] used Hyperledger Fabric to implement an energy metering system with privacy protection for smart grids. Che et al. [25] implemented a prosumer transaction system using Hyperledger Fabric with a focus on transaction authentication mechanisms. These features of Hyperledger Fabric thus, make it particularly suited for developing a peer to peer energy transaction system.

Such a system would involve three main actors/organizations. The Transaction Platform would be the first organization and it would include all the small scale energy prosumers and EV owners. The Power Company that supplies electricity to the community would be the second organization. Finally, the Storage Provider which stores the renewable energy generated by the prosumers would be the third organization in this network. In our previous work [26] we highlighted the need for an energy transaction system for prosumer communities and discussed the applicability of blockchain to build such a transaction system. Subsequently [27], we identified the potential stakeholders in this organization and proposed and defined tokens to encapsulate different types of energy assets.

Assets on the blockchain are represented by tokens in order to facilitate transactions. Tokens are broadly categorized into two categories fungible tokens (FT) and non fungible tokens (NFT), based on whether they are identical and interchangeable or not. FT are interchangeable and identical in all respects and are divisible. On the other hand NFT cannot be substituted for other tokens of the same type and are indivisible. In an energy transaction system, energy assets with an attached Guarantee of Origin [28] have a unique identifier and are not interchangeable and can be implemented as NFT. Conversely, if energy assets are considered interchangeable, then tokens representing them can be broken up and traded in parts and can be implemented as FT. Both implementations are relevant in energy transaction systems.

Implementation of NFT and FT requires defining the lifecycle of the tokens from being issued to being redeemed. Methods and algorithms to take the tokens through the lifecycle need to be created and implemented and any challenges that arise need to be identified and suitably addressed. Comparative analysis of both types of tokens in terms of design, implementation, performance and limitations can provide a guide for future implementations of a transaction system. Mezquita et al. [29] proposed an architecture for transaction of fungible energy transactions on the Ethereum blockchain. However, this work did not feature an implementation of their proposal. Pop et al. [30] proposed a blockchain based peer to peer energy market for NFT focusing on aspects such as prosumer access control, automation of bids and offers matching. However, to the best of the our knowledge, a comparative analysis of the design, performance and limitations of FT and NFT based on an implementation has not been studied.

The motivation of this study is to design, implement and performance test a unified transaction system for energy assets represented as Fungible and Non Fungible Tokens that incorporates all the stakeholders and business relationships into a transparent and decentralized solution in order to address the identified gap in literature. Such a system would enable transactions of energy assets over a decentralized peer to peer network. A transparent system with inherent characteristics of immutability, authentication, access control and provenance tracing as well as the ability to encode business logic into smart contracts would enable automation of transactions and can be adapted for future implementations of microgrid transaction systems. Finally, performance is an essential aspect of a transaction system, which can be characterized based on metrics such as transaction throughput and latency.

Based on the aspects discussed in this section and the motivations outlined, the main contributions of this work include the following:

1. A Transaction Platform that includes the Prosumers and the EV owners, the Power Company and the Storage Provider were identified as stakeholders and represented as the three organizations in this business network. The trading relationships and energy assets that are transacted in this system were outlined. The energy assets were encapsulated in a blockchain token structure with token level consensus policies reflecting the identified stakeholders.
2. The main stages in the lifecycle of all tokens were defined as Create, Bid, Transfer and Redeem. The methods and algorithms in order to take the token through the lifecycle were separately developed for NFT and FT due to the different characteristics of these token implementations.
3. A proof of concept was implemented on the Hyperledger Fabric blockchain platform and the developed algorithms were encoded as smart contracts. Experiments were designed and executed in order to performance test the implementation based on transaction throughput and transaction latency metrics.
4. The limitations of the study, proposed solutions and future avenues for investigation were identified.
5. Based on the experiments and analysis, NFT and FT implementations were critically compared in terms of design, performance and limitations.

The remainder of the work has the following structure: Section 2 presents an overview of the system, describing the design rationale, the system participants and tokens and the relations between them. In Section 3, we delve deeper into the design and implementation of the tokenized energy assets, describe the design requirements and address those requirements by presenting methods and algorithms that will take the token through the lifecycle for both NFT and FT versions. Section 4 describes the experimental infrastructure and presents the execution details of our experiments and the results obtained, as well as a comparative analysis of NFT and FT based on performance and limitations. In Section 5 we present the contributions of our work in the context of related works. Section 6 presents the salient conclusions from this study.

## 2. System Overview

The blockchain network consists of three organizations- the Transaction Platform, the Power Company and the Storage Provider. Each organization can have several client identities. A client identity is used by a registered user of the organization in order to identify themselves and access the network resources. The identity determines a user’s access within the system.

### System Participants and Tokens

Prosumers who want to sell their excess energy or buy energy from other prosumers, as well as EV owners who want to charge their batteries can be registered users of the Transaction Platform organization. As members of the network, prosumers can earn reward tokens for participating in demand response tasks, while EV owners can earn reward tokens for renting out their batteries as temporary storage devices. Moreover, prosumers can also use the Transaction Platform to access the Storage Provider and store or withdraw their excess stored energy.

Power Companies can directly enlist customers in their demand response efforts by offering reward tokens. Incentivization tokens can be offered to the prosumers for accomplishing tasks such as estimating their own consumption accurately in advance. Presenting tasks such as not running the air conditioner on a hot day in a gamified context can increase engagement and be rewarded by Gamification tokens. Representatives of power companies can create and offer Incentivization and Gamification tokens, with associated penalties for reneging, for the prosumers to bid on. We consider bidding upon a token as registering binding interest in the token without negotiating the price or value. If the Power Company has access to the energy stored with the Storage Provider, this may further aid their demand response efforts.

The Storage Provider organization handles all the complexities and tasks of setting up a community level energy storage and abstracts away the details from the prosumers. The Storage Provider can be a separate business or can be owned by the Power Company. Pilot studies on the use of community level batteries for peak shaving are currently underway and in this case, often the community battery is also owned and operated by the Power Company [31].

In this system we consider, six different types of tokens in the system in order to fulfil six different functionalities.

1. EUnit energy tokens for transacting renewable energy between prosumers.
2. EVUnit energy tokens for battery charging transactions between prosumers and EV owners.
3. InUnit reward tokens offered to prosumers by the Power Company for tasks such as consumption estimation.
4. GaUnit reward tokens offered to prosumers by the Power Company for energy flexibility in a gamified context.
5. StUnit energy storage tokens for managing the community level energy storage transactions.
6. EStUnit reward tokens for managing the use of EV batteries as energy storage.

**Figure 1:**
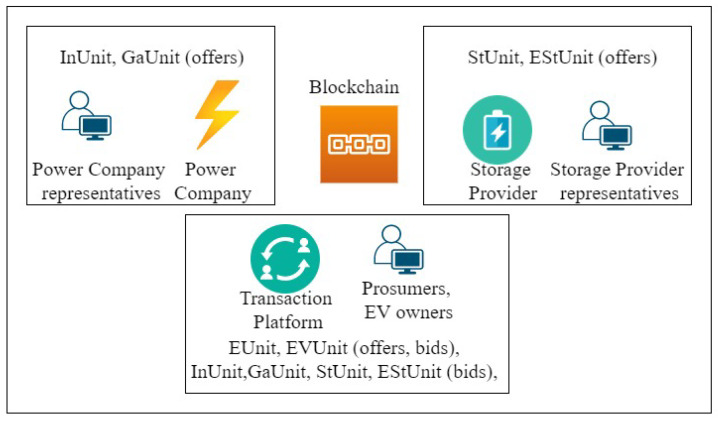
System Participants.

Figure 1 presents an overview of the system participants and the relationships between them.

The implementation of the tokens as FT and NFT is done in two separate chaincodes, written in Golang v1.16 [23], each with smart contracts for functionalities that correspond to the lifecycle of the token, which are create, bid, transfer and redeem. Endorsement policies in Hyperledger Fabric stipulate how many or which organizations must endorse transactions. The chaincode level endorsement policies override the network level endorsement policies. The chaincode level endorsement policies for both of the chaincodes is that two out of the three organizations must endorse each transaction. This chaincode level policy will apply at token creation time for FT as well as NFT implementations. After the token is created, i.e., the key-value pair corresponding to the token is created in the world state, key level endorsement policies for the given token can be configured which will override the chaincode level endorsement policy. For each token, we configure the issuer organization as the organization of the client that invokes the token creation. We also configure a endorser organization for each token that is required to endorse all transactions involving that token. We add both these organizations as required endorsers to each token’s key level endorsement policy. In Table 1, we present a mapping for token types and required issuer and endorsing organizations. We ensure by encoding into the smart contract, that only a client of the stipulated issuer organization is permitted to create a token of the corresponding token type. This mapping is the same for FT and NFT implementations.

As shown in Table 1, for EUnit and EVUnit, the Storage Provider along with the Transaction Platform must endorse each transaction. We envision that the prosumers do not have any local storage and use the community storage to store their energy, and it is from here that the energy is supplied to a buyer. Thus, before being allowed to transact a token, the Storage Provider must check if the energy is actually physically stored for the mentioned amount. Also, the Storage Provider must make sure that the change of ownership occurs successfully at their end and then approve the transaction. Moreover, for StUnit and EStUnit, the Storage Provider as the issuer and the Transaction Platform as the place where the user interacts with these units, must endorse. Similarly, for all transactions involving InUnit and GaUnit, the Power Company and the Transaction Platform must endorse. The Power Company is the issuer and the Transaction Platform is where the prosumers access the system, so both must approve.

## 3. Design and Implementation

**Figure 2:**
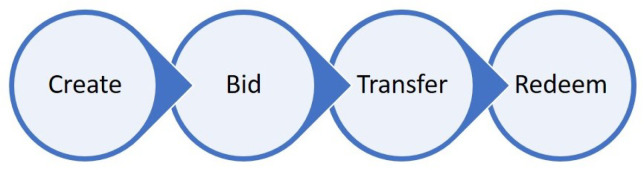
Operations of the token.

A token is acted upon by four operations in its lifecycle as shown in Figure 2. First the token is created by a seller. A buyer then bids upon the token, whereupon the seller transfers the value of token to the buyer. The owner of a token can redeem the value of the token.

### 3.1. Structure of the Token

**Figure 3:**
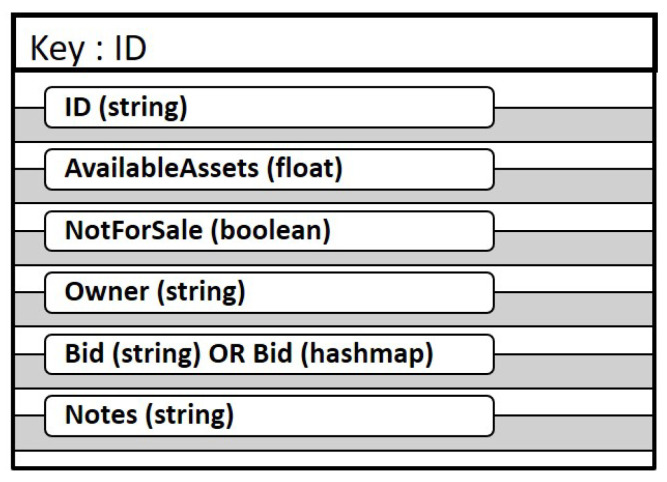
Structure of token.

Each token, whether FT or NFT in our implementation consists of a key value pair where the key is the ID of the token and the value consists of fields with information about the token as shown in Figure 3. However, there are differences in the the design and implementation of the contents of the token fields for FT and NFT and is described in Section 3.3 and Section 3.4.

The fields in the token are:

- *ID* uniquely identifies the token in the network.
- *AvailableAssets* lists the count of energy assets included in this token.
- *Notforsale* is a Boolean flag that is set to TRUE if the token is not for sale.
- *Owner* stores the identity of the owner of the token.
- *Bid* field accepts a bid on the token. This field is implemented as a string in the case of NFT in order to receive a single bid and as a hashmap in case of the FT implementation in order to accept multiple bids. A bid in this system registers a binding interest in a token.
- *Notes* field is a placeholder to store other details of the token such as price or terms and conditions that may be needed in the exact use case but are not general enough for our work.

### 3.2. Design Requirements

The following are the requirements of such a system:

- Methods and algorithms must be provided to take the token through its entire lifecycle with four main operations: create, bid, transfer and redeem.
- The user must be able to query the entire list of all tokens of each type owned by them without having to remember the token ID.
- Only a member of the designated issuer organization of each type of token as defined in Table 1 must be permitted to initiate token creation. Moreover, token level endorsement must be configured on each created token so that the designated endorser organizations of each type of token are required to endorse transactions for that token.
- After the token is created, a designated owner is defined which can be either the issuer or the recipient of the token and only they must be allowed to initiate any transactions to the token. This is with the exception of placing a bid upon the token. For this purpose, another user must be able to access the token and register their bid.
- A user must be able to designate any of their tokens as not for sale and others should not be able to bid upon it.
- When a buyer bids upon a token, their client identity must be recorded, so that a transfer to them can be processed.
- If the token is NFT, then only one bid is permitted on a given token for the entire value of the token.
- For FT tokens, multiple bids are permitted and each must be recorded with the bid amount of energy assets and bidder identity. After each bid on a FT, the value of the available tokens should be updated and bidding should be allowed until all the energy assets in the token have been bid upon. If the same buyer bids multiple times on the same token, the older bid amount must not be lost. The new bid amount must be added to the buyer’s bid.
- In FT tokens, the token must be able to accept further bids on the remaining value, without requiring the owner to transfer the value right away. Moreover, the owner should not have to wait for the entire value of the token to be bid upon before initiating transfer.
- The owner of the token must not be able to spend the token value that has been bid upon. For NFT tokens, the owner of a token with a bid on it must not be able to redeem that token at all. Similarly, for FT tokens, the owner must only be able to redeem value that is still available, i.e has not been bid.

### 3.3. Implementing Energy Assets as NFT

When a NFT is put up for sale, or to be won as a reward, the bidder must bid upon the token in its entirety. This allows the sellers to set different prices for different tokens of the same token type. Also, it allows the Power Company to set up different conditions for tokens of the same token type as per their business need.

LevelDB is used as the state database in this work as it offers better performance than CouchDB which is the other supported state database in Hyperledger Fabric [32]. As mentioned before, the ID field should be unique and each client must be able to query for a list of tokens they own, without having to remember token IDs. However, LevelDB does not support rich querying. So, we accomplish this with the use of range queries in Hyperledger Fabric which takes a start and end value of the ID and returns the list of tokens that have IDs that fall between the values or are equal to the provided values. This means that the client would be expected not only to create IDs having a lexically serialized order, but also remember the first and last token IDs to provide to the query function. If a client were to forget the range to use they would lose access to tokens outside that range. This would be untenable, so our smart contract takes over the task of generating IDs. When creating the the ID of any token, the client identifier and token type must be included in the ID so as to enable range queries allowing the user to query for all tokens they own of a particular type.

In case of NFT, this poses a challenge as multiple tokens can have the same owner and token type, so we need to add in a unique identifier to the ID to distinguish between different tokens each having the same client and token type. We could have saved the maximum value ID used in the system, or per client as a token and refer to that each time a token is created, ensuring that the number is unique across the entire system. Setting a centralized maximum value would need that token to be referred in every single transaction involving that client, creating a bottleneck and slowing down the process. Instead, the transaction ID which uniquely identifies the transaction within the scope of the channel was used and the ID for NFT of is the form:

**ID: clientid_tokentype_transactionID**

#### 3.3.1. Creating a NFT

Algorithm 3 describes the creation of NFTs. The algorithm starts by checking the client’s identity and the identity of the organization of which the client is a part. It then conducts a check to make sure that the invoker organization is permitted to create the requested token type and sets value of the additional organization apart from the invoker that must be added to the endorsement policy. The ID and Owner of the token will depend on whether the token is being created as part of a transfer to a buyer or is being created for the invoker. Algorithm 5 uses Algorithm 3 in order to create a token for a buyer, as will be described in Section 3.3.3. After creating and committing the new token, Algorithm 1 is called to set the key level endorsement policy to override the chaincode level policy. This algorithm accepts the ID of a token in the world state and the organizations that will be included in the new endorsement policy. It instantiates a new policy, adds the requested organizations and commits it.
**Algorithm 1**  SetTokenStateBasedEndorsement1:**function**SetTokenStateBasedEndorsement(id string, issuerorg string, endorserorg string)2:     endorsementPolicy←statebased.NewStateEP()3:     endorsementPolicy.AddOrgs(statebased.RoleTypePeer,issuerorg,endorserorg)4:     policy←endorsementPolicy.Policy()5:    SetStateValidationParameter(id,policy)6:**end function**

**Algorithm 2** ReadToken
1:**function**ReadToken(id string)2:     tokenJSON←GetState(id)3:     token←json.Unmarshal(tokenJSON)4:     return token5:
**end function**



**Algorithm 3** Create a NFT
1:**function**CreateNFT(tokentype string, creatingfortransfer bool, buyer string, availableassets int)2:     invokerorg←GetClientIdentity().GetMSPID()3:     invokerclient←GetClientIdentity().GetID()4:     test←tokentype+“_”+invokerorg5:     **switch test**6:           
“EUnit_Org1MSP”or“EVUnit_Org1MSP”7:           
endorserorg=“Org3MSP”8:           
“InUnit_Org2MSP”or“GaUnit_Org2MSP”9:           
endorserorg=“Org1MSP”10:         
“StUnit_Org3MSP”or“EStUnit_Org3MSP”11:         
endorserorg=“Org1MSP”12:    **Default: “Invoker Organization and Token Type combination invalid”**13:    **if** creatingfortransfer == true **then**14:        idval←buyer+“_”+tokentype+“_”+GetTxID()15:        owner←buyer16:    **else**17:        idval←invokerclient+“_”+tokentype+“_”+GetTxID()18:        owner←invokerclient19:    **end if**20:    tokennew←NewToken(ID←idval,TokenType←tokentype, AvailableAssets ←availableassets,Owner←owner)21:    tokenJSON←json.Marshal(tokennew)22:    PutState(idval,tokenJSON)                                                                                                    ▹ Saving new token23:    SetTokenStateBasedEndorsement(tokennew.ID,invokerorg,endorserorg)                                                                                                   ▹ Calling Algorithm 124:
**end function**



#### 3.3.2. Bidding on a NFT

Algorithm 4 illustrates how a buyer can bid upon a token of their choosing. The requested token is read using Algorithm 2 and checked if it is for sale and that there is no bid on this token already. If there is no bid, then the algorithm updates the bid field with the client identity of the buyer and commits it to state. As the token must be purchased in its entirety, there is no mention of the amount of energy assets in the bid.
**Algorithm 4** Bid on a NFT1:**function**BidNFT(id string)2:     token←ReadToken(id)                            ▹ Calling Algorithm 23:     **if** token.NotForSale == true **then**4:         return “Token ID not for sale”5:     **end if**6:     bidderclient←GetClientIdentity().GetID()7:     **if** token.Bid==“” **then**8:           token.Bid=bidderclient9:     **else**10:         return “There is already a bid on this token by token.Bid”11:   **end if**12:   tokenJSON←json.Marshal(token)13:   PutState(id,tokenJSON)                                             ▹ Saving updated token14:**end function**

#### 3.3.3. Transferring a NFT

Using Algorithm 5 the owner of a token can transfer the token to the buyer who has bid upon it. The token is read using Algorithm 2 and it is checked that the invoker client is in fact the owner of the token and there exists a bid on the token. If the token exists, is bid upon and the transfer is requested by the owner, the transfer can take place. We will need the newly transferred token to be reflected when the new owner checks their list of tokens using Algorithm 7 which will use the ID of the token stored on the world state. However, we cannot actually edit the ID of the token on the world state, so we create a new token owned by the buyer for the same value using Algorithm 3 and destroy the original token owned by the seller.
**Algorithm 5** Transfer a NFT1:**function**TransferNFT(id string)2:     token←ReadToken(id)                            ▹ Calling Algorithm 23:     invokerclient←GetClientIdentity().GetID()4:     **if** invokerclient != token.Owner **then**5:           return “The client invokerclient is not authorized to transfer token owned by token.Owner”6:     **end if**7:     **if** token.Bid==“” **then**8:           return “No bid yet”9:     **end if**10:   CreateNFT(token.TokenType,TRUE,token.Bid,token.availableassets)                         ▹ Calling Algorithm 3, to create new token with buyer’s id11:   DelState(id)                               ▹ Delete the token with seller’s id12:**end function**

#### 3.3.4. Redeeming a NFT

When the token is redeemed it is deleted from the world state, but the record of transactions remain in the ledger. The Algorithm 6 for redeeming a token, starts by getting the invoker client’s identity to make sure they are the owner of the token being redeemed. Also, a owner should not be able to redeem a token that already has a bid, so the algorithm checks to make sure there is no bid on the token being redeemed. If a token with no bid is being redeemed by the owner, the redeem operation goes through as requested.
**Algorithm 6** Redeem a NFT1:**function**RedeemNFT(id string)2:     token←ReadToken(id)                  ▹ Calling Algorithm 23:     invokerclient←GetClientIdentity().GetID()4:     **if** invokerclient != token.Owner **then**5:           return “The client invokerclient cannot redeem token owned by token.Owner”6:     **end if**7:     **if** token.Bid != nil **then**8:           return “The client invokerclient cannot redeem token as it has a bid”9:     **end if**10:   DelState(id)                                 ▹ Deleting token11:**end function**

#### 3.3.5. Querying for a List of Owned NFTs

As mentioned in Section 3.2, an important design consideration is that the user must not be required to remember the IDs of all the tokens they own and the system must provide an easy way to query that. Algorithm 7 uses a range query to return a list of all tokens of the specified type owned by the invoking client. The algorithm takes the token type queried for as input and gets the invoker client’s ID to create start and end values for the range query by padding to the right to create alphanumeric strings of the same length as the transaction ID in order to get the smallest and largest possible transaction IDs. This returns an iterator which loops through to produce the list of tokens to be returned to the user.
**Algorithm 7** Get my NFT1:**function**GetMyNFT(tokentype string)2:     ownerclient←GetClientIdentity().GetID()3:     checkstr←ownerclient+“_”+tokentype+“_”4:     resultsIterator←GetStateByRange(checkstr+pad(0,64),checkstr+pad(z,64)5:     defer resultsIterator.Close()6:     var tokens []*Token7:     **for** resultsIterator.HasNext() **do**8:          queryResponse←resultsIterator.Next()9:          token←json.Unmarshal(queryResponse.Value)10:        tokens←append(tokens,token)11:    **end for**12:    return tokens              ▹ Returns all tokens of tokentype for requesting client13:**end function**

### 3.4. Implementing Energy Assets as FT

FT are those that are for all intents and purposes identical. Thus they can be broken up and traded in parts, and added up like currency. Each client will have at most six tokens, one of each type of tokens EUnit, EVUnit, InUnit, GaUnit, StUnit and EStUnit. Additional tokens, whether created or purchased will be added to this token value. Tokens redeemed or sold will be reduced from the token value. Thus tokens for a given client in the FT implementation act as accounts, where each client has at most six accounts. The ID of the token in the FT implementation is of the form:

**ID: clientid_tokentype**

Also, as mentioned in Section 3.1, in the FT implementation a token can have multiple bids from the same or different buyers. In order to accomplish this we implemented the Bid field as a hashmap. The hashmap stores a list of key value pairs where key stores the identity of the bidder and the value stores the bid amount. The Bid field thus takes bids on the token and keeps adding bids to the hashmap. If the same bidder bids multiple times on a token, the value for that client key is updated with the total of the bids.

#### 3.4.1. Create a FT

Algorithm 8 for creating a FT first verifies that the invoking client is from an organization that is allowed to create the requested type of token.

Additionally, it sets the appropriate additional organization needed to endorse this token as described in Table 1. The token ID depends on whether the token is being created for the invoker or for transfer to a buyer. Next, the algorithm will check if the token exists by trying to read it. As this is a FT implementation, if the token exists already, a new token will not be created but the requested energy asset count will be added to the existing token. If it is not found, this means that the invoker (if creating for self) or buyer (if creating for transfer) does not already own a token of this type. If so, a new token is created with the appropriate ID, supplied token type and energy asset count and added to the world state. Moreover, the applicable key level endorsement policy is set using Algorithm 1.
**Algorithm 8** Create a FT1:**function**CreateFT(tokentype string, creatingfortransfer bool, buyer string, availableassets int)2:     invokerorg←GetClientIdentity().GetMSPID()3:     invokerclient←GetClientIdentity().GetID()4:     test←tokentype+“_”+ownerorg5:     **switch test**6:          
“EUnit_Org1MSP”or“EVUnit_Org1MSP”7:          
endorserorg=“Org3MSP”8:          
“InUnit_Org2MSP”or“GaUnit_Org2MSP”9:          
endorserorg=“Org1MSP”10:        
“StUnit_Org3MSP”or“EStUnit_Org3MSP”11:        
endorserorg=“Org1MSP”12:   **Default: “Invoker Organization and Token Type combination invalid”**13:   **if** creatingfortransfer == true **then**14:        id←buyer+“_”+tokentype15:        owner←buyer16:    **else**17:        id←invokerclient+“_”+tokentype18:        owner←invokerclient19:    **end if**20:    token←ReadToken(id)                                          ▹ Calling Algorithm 221:    **if** token!=nil **then**22:        token.AvailableAssets←token.AvailableAssets+availableassets23:        tokenJSON←json.Marshal(token)24:        PutState(id,tokenJSON)25:     **else**26:        tokennew←NewToken(ID←id,TokenType←tokentype, AvailableAssets ←availableassets,Owner←owner)27:        tokenJSON←json.Marshal(tokennew)28:        PutState(id,tokenJSON)29:        SetTokenStateBasedEndorsement(tokennew.ID,ownerorg,endorserorg)                                                       ▹ Calling Algorithm 130:    **end if**31:**end function**

#### 3.4.2. Bid on a FT

Algorithm 9 for bidding on a FT accepts the ID of the token as well as the number of energy assets in the bid. It reads the token using Algorithm 2 and checks if the token is available for sale and that the number of energy assets in the bid do not exceed the value of the token. If the same buyer has bid on the token before, the new bid value is added to the old bid value in the existing key value pair of the hashmap. If this is a new buyer, a new key value pair is created in the hashmap to accept the bid, where the key is the client identity and value is the amount of the bid. Finally, the count of available assets in the token is reduced by the amount of the bid and the updated token is committed to the state.
**Algorithm 9** Bid on a FT1:**function**BidFT(id string)2:     token←ReadToken(id)                                    ▹ Calling Algorithm 23:     **if** token.NotForSale==true **then**4:            return “Token ID is not for sale”5:     **end if**6:     **if** token.AvailableAssets<bidvalue **then**7:            Return “Available value token.AvailableAssets is less than bid bidvalue”8:     **end if**9:     bidderclient←GetClientIdentity().GetID()10:     **if** token.Bidmap[bidderclient]!=nil **then**11:          token.Bidmap[bidderclient]←existingval+bidvalue12:    **else**13:        token.Bidmap[bidderclient]=bidvalue14:    **end if**15:    token.AvailableAssets=token.AvailableAssets−bidvalue16:    tokenJSON←json.Marshal(token)17:    PutState(id,tokenJSON)                                   ▹ Saving updated token18:**end function**

#### 3.4.3. Transfer a FT

Algorithm 10 for transferring a FT, first reads the token using Algorithm 2 and verifies that the invoker client is the owner of the token being transferred. Then for each bid in the bid hashmap, it processes the value transfer to the buyer, using Algorithm 8. If the token for the buyer exists already, it is updated with the transferred value. Otherwise a new token is created for the buyer and a key level endorsement policy is set. The processed bid is now removed from the map.

When all the bids have been processed and deleted, finally, it updates the token that was being transferred with the newly empty bid field into the state. As the value of available energy assets on the token is reduced by the value of the bid whenever a bid is placed, as explained in Section 3.4.2, the count of available assets on the token already reflects the value after deducting the bid amounts.
**Algorithm 10** Transfer a FT1:**function**TransferFT(id string)2:     token←ReadToken(id)                                                           ▹ Calling Algorithm 23:     invokerclient←GetClientIdentity().GetID()4:     **if** invokerclient != token.Owner **then**5:          return “The client invokerclient is not authorized to transfer token owned by token.Owner”6:     **end if**7:     **for** key,value←range(token.Bidmap) **do**8:           CreateToken(token.TokenType, TRUE, key, value)                                              ▹ Calling Algorithm 89:           delete(token.Bidmap,key)10:    **end for**11:    tokenJSON←json.Marshal(token)12:    PutState(id,tokenJSON)                                                  ▹ Save updated token after transfers13:**end function**

#### 3.4.4. Redeem a FT

Tokens in the FT implementation work like accounts, so when a FT is redeemed, some or all of the available energy assets may be redeemed, but the token itself not deleted as is the case for the NFT implementation described in Section 3.3.4. The Algorithm 11 for redeeming FT begins by reading the token using Algorithm 2 and checking that the invoker client owns the token and that the requested redeem amount is not greater than the total available energy assets in the token. As the available assets count is reduced by the bid amount for every bid on the token as described in Section 3.4.2, it reflects the true count of the assets available to the token owner for redeeming. Next, the available energy assets on the token is reduced by the redeem value and the updated token is then committed to the world state.
**Algorithm 11** Redeem a FT1:**function**RedeemFT(id string, redeemcount int)2:     token←ReadToken(id)                                    ▹ Calling Algorithm 23:     invokerclient←GetClientIdentity().GetID()4:     **if** invokerclient!=token.Owner **then**5:           return “The client invokerclient is not authorized to redeem token token.ID owned by another”6:     **end if**7:     **if** redeemcount>token.AvailableAssets **then**8:           return “The token token.ID has token.AvailableAssets assets, which is less than requested redeem value redeemcount”9:     **end if**10:   token.AvailableAssets←token.AvailableAssets−redeemcount11:   tokenJSON←json.Marshal(token)12:   PutState(id,tokenJSON)13:**end function**

#### 3.4.5. Querying for a List of Owned FT

For any client there will be six FT at most, one corresponding to each of the types of tokens in the system which are EUnit, EVUnit, GaUnit, InUnit, StUnit and EStUnit. The Algorithm 2 provides the list of FT of the specified token type owned by the invoking client.

### 3.5. Complexity of the Algorithms

The complexity of an algorithm describes its efficiency in terms of the size of the input. The two main complexity measures of the efficiency of an algorithm are time and space complexity. Time complexity describes the time taken to execute an algorithm independently of factors that are unrelated to the algorithm. Factors such as programming language, memory cache, type of processing capacity and compiler optimization are not related to the algorithm itself but can affect the actual time taken to execute the algorithm. Similarly, space complexity describes the amount of memory space needed to execute an algorithm independently of the actual hardware used.

NFT has operations, NFT Create (Algorithm 3), NFT Bid (Algorithm 4), NFT Transfer (Algorithm 5), NFT Redeem (Algorithm 6) and NFT BulkRead (Algorithm 7). Similarly, FT has the operations FT Create (Algorithm 8), FT Bid (Algorithm 9), FT Transfer (Algorithm 10) and FT Redeem (Algorithm 11).

In Algorithm 8 for FT, if a token exists already, then the token will simply be updated and the key level endorsement policy will not be set again. In order to distinguish between the two types of create operations in FT, we call the operation FT ReCreate if the token is updated and the endorsement policy is not set again. If the token is created and the key level endorsement policy is configured, we call it FT Create. Similarly, for the Algorithm 10, we have two operations FT Transfer, if the token is created and FT ReTransfer if the token is updated with the transferred value.

Table 2 shows the time and space complexities of all the algorithms described in Section 3.3 and Section 3.4.

In Hyperledger Fabric, create, update, endorsement policy configurations and deletes are all processed as writes to the state. Thus, NFT Create involves no read operations and two write operations, one to create the token and one to configure the endorsement policy. Also, this operation only uses space to store that one token. The time and space complexities for this algorithm are O(1), as for any invocation, only one token will be created using the mentioned operations. Similarly, NFT Bid (one read, one write), NFT Transfer (one read, three writes) NFT Redeem (one read, one write), NFT Read (one read), FT Create (one read, two writes), FT ReCreate (one read, one write), FT Bid (one read, one write), FT Redeem (one read, one write) and FT Read (one read) will have a constant number of operations as well as space usage for any invocations and thus have both time and space complexities of O(1).

NFT BulkRead will execute read operations, which will depend on and grow with the number of tokens owned by the invoking client. Here, each token returned by the range query is considered to be a separate read. Similarly, the space used will also depend on the number of tokens returned. Thus, the time and space complexities of NFT BulkRead are both O(n).

FT Transfer and FT ReTransfer will execute one read in order to read the seller’s token. Then, both operations will execute a loop based on the number of bids present. For each bid, they will execute one read to check if the buyer’s token exists and one write to either create (FT Transfer) or update (FT ReTransfer) the buyer’s token. FT Transfer will execute an additional write in order to set the key level endorsement policy for the buyer’s token. In each execution of the loop, both operations will use the space required to operate upon two tokens, the buyer’s token and the seller’s token. As the number of times this loop is run will depend on the number of bids present, the time and space complexities of both FT Transfer and FT ReTransfer are O(n). Finally, at the end of the loop, the seller’s token will be updated with a single write, which is constant for any invocation.

Multiple transfers that originate from a single FT perform transfer of value to multiple distinct recipients in a FT Transfer operation. In order to complete comparable transfers in NFT, we would need to perform multiple separate transactions, one for each bid. Thus, even though the time and space complexities of the presented algorithms for FT Transfer and ReTransfer are O(n), we do not consider them to be a bottleneck as they cannot be considered equivalent to a single NFT transfer. A fair comparison, between the value transfer operations Transfer and ReTransfer for FT and Transfer for NFT, should thus consider a single bid per token. We performance test and make a comparison between these three algorithms in Section 4.2.

For NFT, retrieving all tokens of a type owned by a client is expected to be a bottleneck if executed on chain. We experimentally investigate this in Section 4.2 and the results of our experiments are shown in Figure 10.

### 3.6. Token Lifecycle

**Figure 4:**
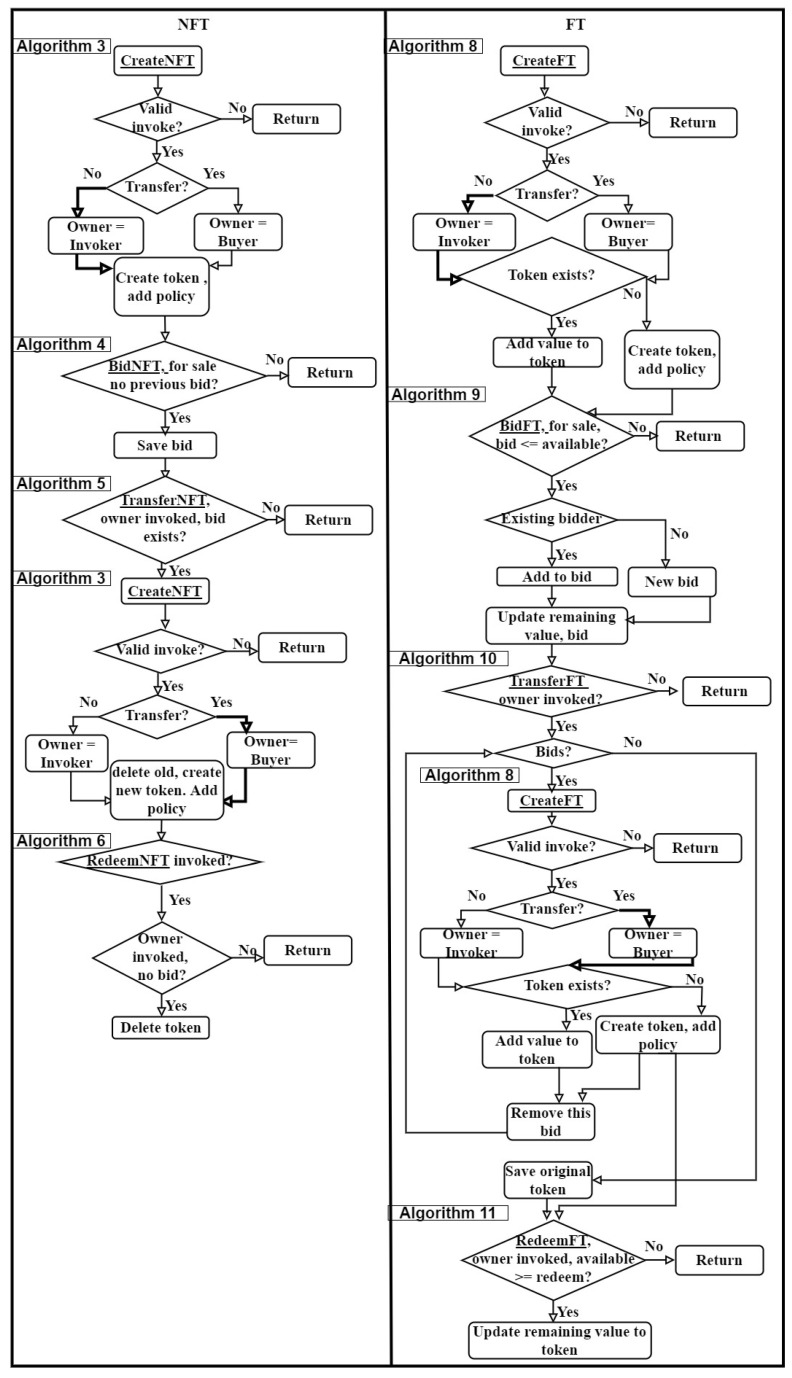
Lifecycle for Fungible Tokens (FT) and Non Fungible Tokens (NFT).

**Figure 5:**
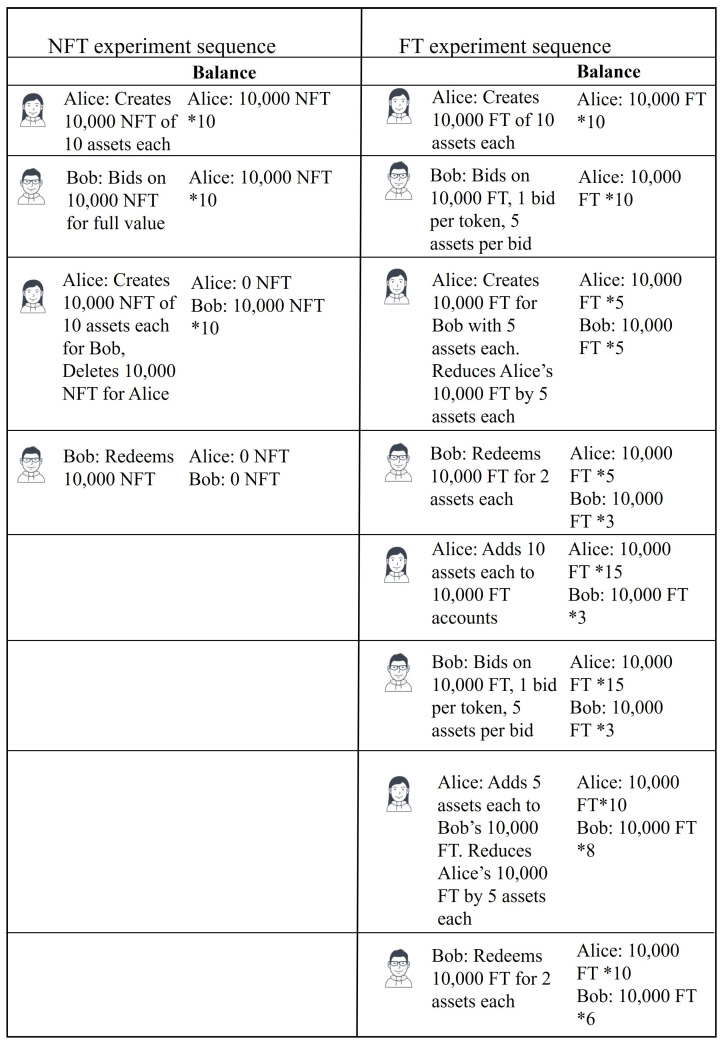
Sequence of experiments.

The algorithms developed for managing token lifecycles for both NFT and FT, have been described in detail in Section 3.3 and Section 3.4 respectively. The overall token lifecycle and the associated algorithms called at different stages from create to redeem have been summarized as flowcharts in Figure 4. The sequence of experiments shown later on in Figure 5 were developed based on this workflow in order to performance test and compare the implementations of the specific algorithms.

The left pane in Figure 4 shows the lifecycle of a NFT. First, the seller invokes Algorithm 3 (Section 3.3.1) to create the token. If the invoke is valid, a check is performed to see if the token is being created for a transfer to a buyer. At this stage in the lifecycle, the token is being created for the invoker so the execution path highlighted in the flowchart applies and the invoker is the owner of the token. The token is then created and the token level endorsement policy is set. The token is now ready to accept a bid. The buyer calls Algorithm 4 (Section 3.3.2) and if the token is for sale and no previous bid is present, the bid is accepted. The seller then calls Algorithm 5 (Section 3.3.3), to transfer the token to the buyer who has bid upon it. In order to do so, a new token is created for the buyer by calling Algorithm 3 and executing the path highlighted in the flowchart. This new token has no bid on it. The original token owned by the seller is deleted. The current owner of the token can redeem the token using Algorithm 6 (Section 3.3.4) if there is no existing bid on it. A redeemed NFT is deleted from the state.

The right pane of Figure 4 summarizes the lifecycle of a FT. First, Algorithm 8 (Section 3.4.1) is invoked by the seller. At this stage of the lifecycle, the token is being created for the invoker. If the invoke is valid, a check is performed to see if a token for the same token type and owner combination exists. If such a token exists, the value of the token to be created is added to the value of the existing token and no new token is created. Otherwise, a new token with a token level endorsement policy is created. This token is now ready to accept bids. A buyer uses Algorithm 9 (Section 3.4.2) in order to bid upon the token. If the same bidder has already bid upon the token, the value of the new bid is added to the existing bid, else a new bid is accepted on the token. In contrast to a NFT, a FT can accept multiple bids on the available value, and after each bid the value of the token is reduced by the bid amount. The seller, using Algorithm 10 (Section 3.4.3), for each bid will create a new token for the buyer using Algorithm 8 and remove the bid from the original token. This will be done until all the bids are removed from the token. A token can be redeemed by its owner for the value available in the token. Thus, the buyer can redeem the newly purchased token and the seller can redeem the value left on their token using Algorithm 11 (Section 3.4.4). A FT that is redeemed is not deleted, but the value of the token is reduced by the redeem amount.

## 4. Experimental Setup, Results and Discussion

The experimental infrastructure included 5 Virtual Machines (VM) created on a cloud environment. Each VM used Ubuntu 20.04 and had 32 GB RAM, 4 dedicated virtual CPUs and a 100 GB SSD. The nodes of the network were implemented as Docker containers with Docker version 19.03 and Docker Compose version 1.26 connected in a Docker Swarm for availability. Hyperledger Fabric v2.3.0 was the blockchain platform and Hyperledger Caliper v 0.4.2 was used to generate the load and measure the performance. Both are the latest stable versions at the time of writing. The Ordering service was implemented using the RAFT [33] consensus algorithm and had 3 Ordering Service Nodes (OSN) as recommended in the Hyperledger Fabric official documentation [34]. LevelDB was the state database due its performance advantage. The three organizations in our network are implemented with one peer node each and run on separate VMs. One VM is dedicated to running the Hyperledger Caliper and another VM runs the Ordering Service which is implemented as a separate Orderer Organization. In a production implementation, cloud security issues and mitigation strategies would need special consideration. Singh et al. [35] conducted an extensive survey of specific threats and solutions to be considered for better security management for a cloud based service.

### 4.1. Experimental Setup

In order to performance test the implementation of each algorithm, the sequence of experiments shown in Figure 5 were designed based on the token lifecycle shown in Figure 4 and explained in Section 3.6, for NFT and FT algorithms.

A client, Bob was created for the Transaction Platform and another client Alice was the created for Storage Provider. The left pane on Figure 5 shows the sequence of operations for experimental evaluation of the NFT implementation. Using Algorithm 3 Alice creates 10,000 NFT each with value of 10 assets. Bob bids upon each of these tokens using Algorithm 4 and as this is a NFT implementation, the bids are for the whole value of the asset. Alice then initiates transfer to Bob using Algorithm 5, which involves creating 10,000 NFT owned by Bob each with the value of 10 assets and deleting all 10,000 NFT owned by Alice. Finally, using Algorithm 6, Bob redeems the complete value of all 10,000 NFT.

Similarly in the FT implementation, clients Alice and Bob are created for the Storage Provider and the Transaction Platform respectively. The sequence of operations for experimental evaluation of the FT implementation is shown in the right pane of Figure 5. In order to have a fair comparison between FT and NFT implementations, and because it was not feasible to implement thousands of distinct clients in order to create 10,000 tokens of the predefined token types, Alice creates 10,000 separate FT with 10 assets each using Algorithm 8, effectively creating 10,000 accounts owned by Alice. Bob places one bid per FT with 5 assets per bid on all 10,000 FT using Algorithm 9. Alice initiates transfer using Algorithm 10 which creates 10,000 FT for Bob, each with value 5 assets and reduces the value of Alice’s FT by the bid amount, in this case 5 assets. Bob redeems 10,000 FT for 2 assets each using Algorithm 11 leaving a balance of 10,000 FT with 3 assets each for Bob. Alice’s balance at this point is 10,000 FT with 5 assets each.

Alice again initiates creation of 10,000 FT with the same IDs as before with 10 assets each using Algorithm 8. This time, however, as the FT already exist, the created value is added to the existing FT. In order to distinguish this operation from the create operation executed before, we call this ReCreate. Bob again bids on all 10,000 FT with 1 bid per FT and 5 assets per bid using Algorithm 9. Alice initiates transfer using Algorithm 10. Bob’s FT exist already, as they were created before. So, the algorithm adds the value of the bid to Bob’s FT making Bob’s balance 10,000 FT with 8 assets each. Alice’s balance after transfer is 10,000 FT with 10 assets each. In order to distinguish this from the transfer done before, we call this ReTransfer. Finally, Bob redeems all 10,000 FT for the value of 2 assets each leaving a balance of 10,000 FT with 6 assets each.

In case of FT, the number of key operations in each operation depend on the number of bids received on each token as explained in Section 3.5. However, for a fair comparison, for both NFT and FT, we place only one bid on each token as described above and in the right pane of Figure 5. Hyperledger Caliper was used to drive the load and measure performance as mentioned before. The load was driven with four worker processes and the fixed load rate control mechanism was configured. The fixed load rate controller starts with a configured transaction send rate in transactions per second (TPS) and maintains a defined queue length of unfinished transactions in the network by modifying the send rate. The starting send rate was set to 1000 TPS for all our experiments, while the queue length was varied. The sequence of operations between Alice and Bob described above was conducted 10 times each for NFT and FT implementations by varying the queue length from 100 unfinished transactions to 1000 unfinished transactions with equal increments of 100 TPS. In addition, experiments were conducted on the Read operation described in Algorithm 2 for both NFT and FT with 1 read per query and 10,000 queries in one iteration, with a total of 4 iterations with queue lengths varying from 100 unfinished transactions to 400 unfinished transactions with a step size of 100. Increasing the queue length beyond this point did not show any increase in throughput. Moreover, in case of NFTs, each client can have several tokens, which can be retrieved using Algorithm 7. In order to test this algorithm, experiments were conducted for the bulk read operation by running 4 iterations with 10,000 bulk read queries in one iteration with each bulk read query returning 10,000 NFT. The queue lengths were varied from 100 to 400 over the 4 iterations with a step size of 100. Queue lengths of more than 400 unfinished transactions did not result in any improvement in throughput. Table 3 shows a summary of key transactions such as read, write and key level endorsement policy configuration that were involved in each operation conducted in the experimental evaluation.

### 4.2. Results and Discussion

An endorsing peer simulates each transaction before endorsing and creates a read-write set. Deletes are processed by setting a delete marker in the write set. Similarly, key level endorsements, creation and updating of key value pairs are also processed as writes to the state in the read-write set. Thus for performance analysis, we consider them all as writes to the state.

**Figure 6:**
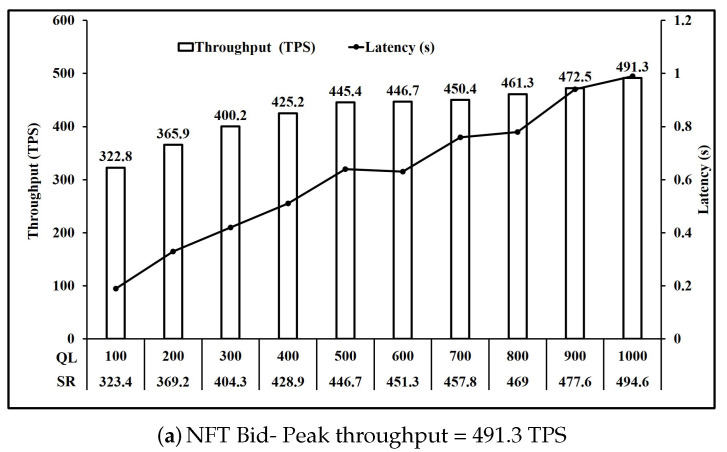

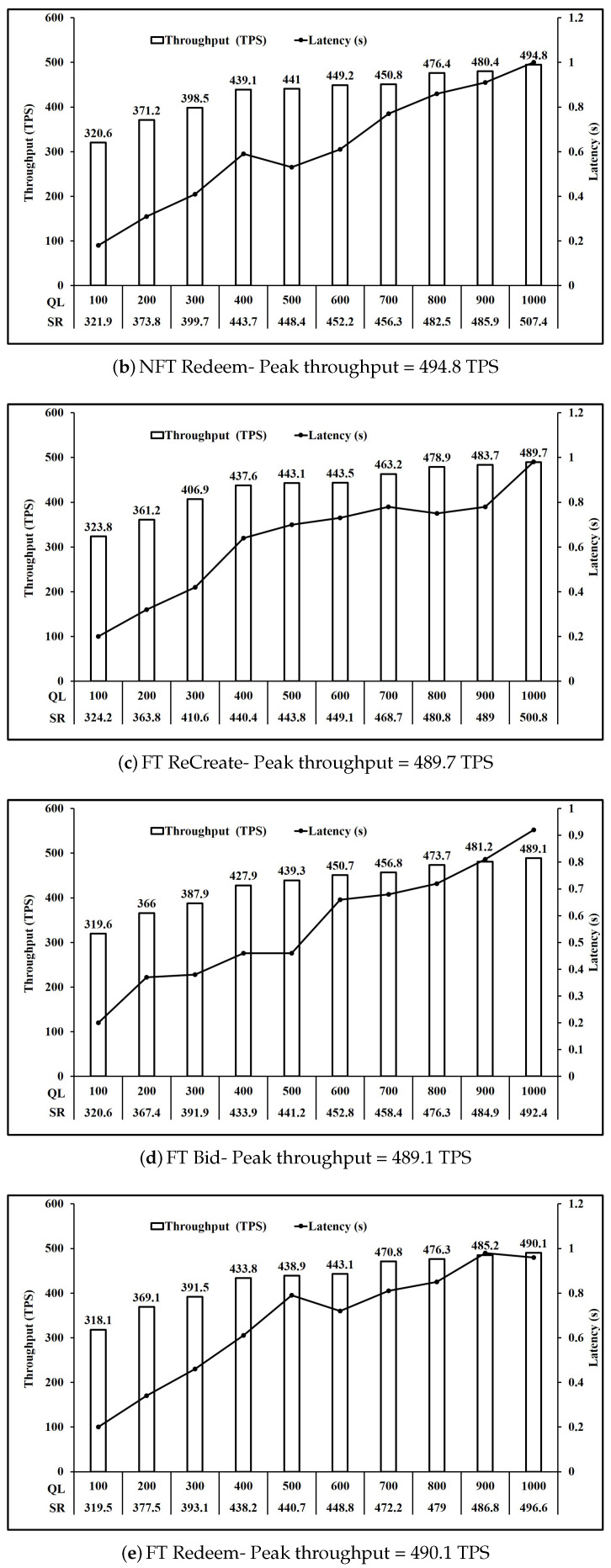
Operations with one write key operation (Queue Length: QL, Send Rate (TPS): SR).

**Figure 7:**
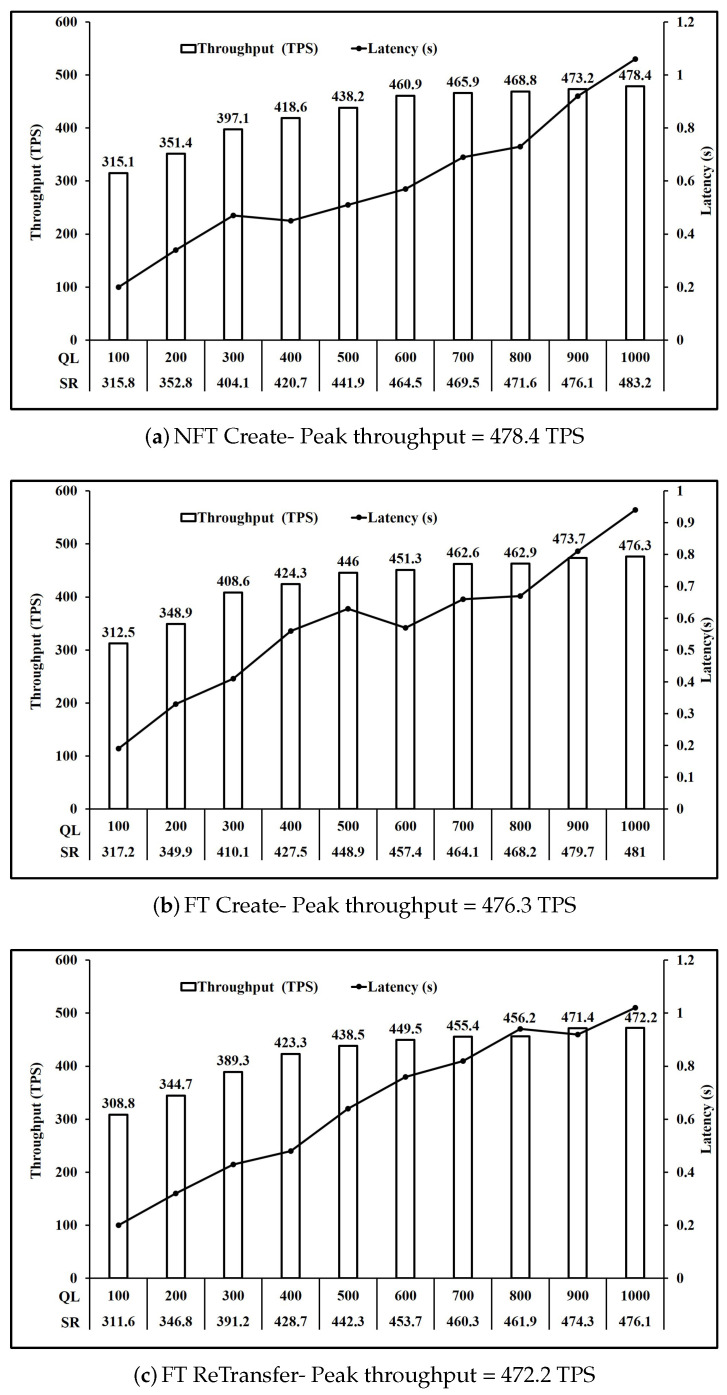
Operations with two write key operations (Queue Length: QL, Send Rate (TPS): SR).

**Figure 8:**
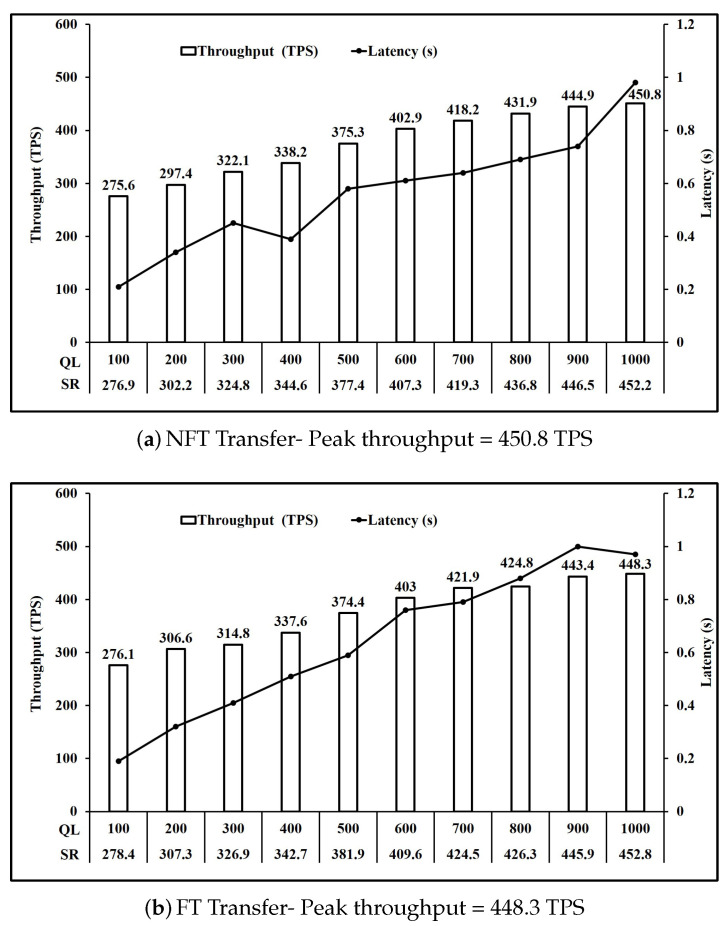
Operations with three write key operations (Queue Length: QL, Send Rate (TPS): SR).

**Figure 9:**
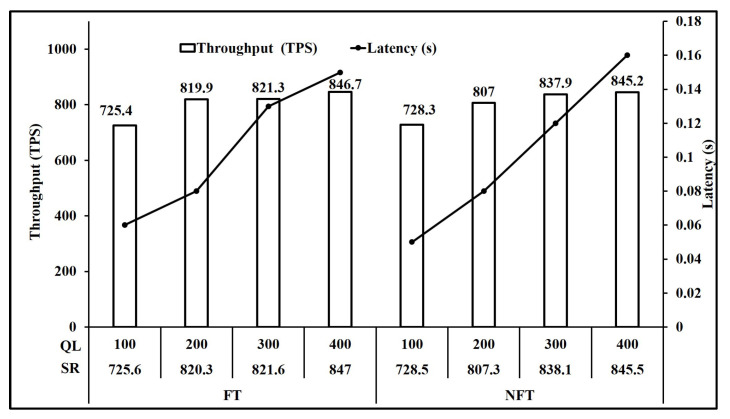
Read operation- Peak Throughput: FT- 846.7 TPS, NFT- 845.2 TPS (Queue Length: QL, Send Rate (TPS): SR).

For our experiments, we define transaction complexity of an operation as the number of writes to the state executed in that operation. Based on Table 3, we can group the operations based on transaction complexity. Figure 6 shows the performance of operations involving one write key operation. Here, we observe that all five operations NFT Bid, NFT Redeem, FT ReCreate, FT Bid and FT Redeem have comparable peak throughput of 491 TPS on average. Similarly, Figure 7 and Figure 8 present the performance of operations involving two and three write key operations respectively. It is seen in Figure 7 that all three operations NFT Create, FT Create and FT ReTransfer have comparable peak throughput of 475.6 TPS on average. Also, Figure 8 shows that operations NFT Transfer and FT transfer also have comparable peak throughput of 449.55 TPS on average. Performance of read operations for FT and NFT shown in Figure 9 also show a comparable peak throughput of 845.95 TPS on average for both NFT and FT implementations.

**Figure 10:**
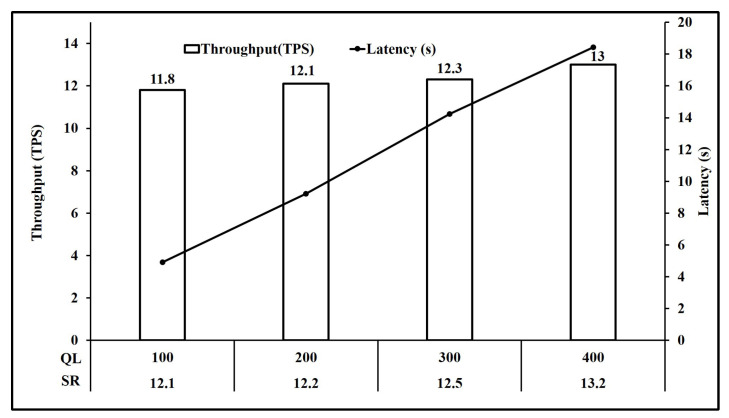
NFT Bulk Read Operation. Peak Throughput 13 TPS (Queue Length: QL, Send Rate (TPS): SR).

As mentioned in Section 4.1, we used the fixed load rate controller in Hyperledger Caliper which maintains the configured queue length by modifying the send rate. Figure 6a shows that when the queue length is increased from 100 unfinished transactions to 1000 unfinished transactions, the request send rate achieved rose from 323.4 to 494.6. Moreover, the throughput achieved rose from 322.8 TPS to 491.3 TPS at a cost to the per transaction latency which also rose from 0.19 s to 0.99 s. Similar observations can be made for all operations in Figure 6, Figure 7, Figure 8, Figure 9 and Figure 10.

Comparing Figure 6 and Figure 7, we see that the throughput achieved reduces from 491 TPS on average to 475.6 TPS on average when the number of writes per transaction increased from 1 to 2. Similarly, comparing Figure 7 and Figure 8, we see that the throughput achieved further reduces to 449.55 TPS on average when number of writes per transaction increased from 2 to 3.

Analysis of Figure 7a,b shows that a similar performance is achieved for Create operation FT and NFT, as the throughput achieved is 476.3 TPS and 478.4 TPS respectively. Moreover, FT Bid in Figure 6d and NFT bid in Figure 6a are 489.1 TPS and 491.3 TPS respectively. Similarly, Transfer operation throughput for FT shown in Figure 8b and NFT shown in Figure 8a are 448.3 TPS and 450.8 TPS respectively. Finally Redeem operation throughput for FT shown in Figure 6e and NFT shown in Figure 6b are similar at 490.1 TPS and 494.8 TPS respectively. Read operation throughput for FT and NFT both shown in Figure 9 are 846.7 TPS and 845.2 TPS respectively. Thus, for the Create, Bid, Transfer, Redeem and Read, FT and NFT show similar performance for the same number of tokens.

However, FT operations ReCreate and ReTransfer, do not set the key level endorsement policy, as tokens with their associated key level endorsement policy already exist as compared to Create and Transfer for FT and NFT. This is reflected in the performance as ReCreate as shown in Figure 6c achieved throughput of 489.7 TPS compared to 476.3 TPS for FT Create as shown in Figure 7b and 478.4 TPS for NFT Create as shown in Figure 7a. Similarly, ReTransfer throughput shown in Figure 7c at 472.2 TPS was higher than 448.3 TPS for FT Transfer shown in Figure 8b and 450.8 TPS for NFT transfer shown in Figure 8a.

For NFT, Bid (Figure 6a) and Redeem operations (Figure 6b) were the fastest at 491.3 TPS and 494.8 TPS respectively, followed by Create (Figure 7a) at 478.4 TPS and then by Transfer (Figure 8a) at 450.8 TPS. For FT Bid (Figure 6d), Redeem (Figure 6e) and Recreate (Figure 6c) were the fastest at 489.1 TPS, 490.1 TPS and 489.7 TPS respectivly. Create (Figure 7b) and ReTransfer (Figure 7c) were slower at 476.3 TPS and 472.2 TPS respectively, while Transfer (Figure 8b) at 448.3 TPS was the slowest operation for FT.

Finally, Figure 10 shows the results of performance testing the implementation of algorithm 7 for retrieving all NFT owned by a client by performing bulk read operations. As mentioned in Table 3, each operation involves bulk reads of 10,000 NFT. As expected the performance achieved is poor. The peak throughput achieved in our experiments was 13 TPS for a latency of 18.44 s at the queue length of 400 unfinished transactions. Thus, the execution of Algorithm 7 should be considered to be handled off-chain as is mentioned in the Hyperledger Fabric official documentation [36].

### 4.3. Comparison of Non Fungible Tokens and Fungible Tokens

An advantage of the NFT implementation is that it allows the seller of energy assets to set different prices for different tokens of the same token type. Similarly, this implementation permits the actor offering rewards to set different conditions for rewards of the same type. However, in cases where standardization of assets is a requirement, the FT implementation offers an advantage as it ensures uniformity between tokens of the same token type.

FT maintain an updated count of tokens for each token type for each client in a single token, so that each client has at most 6 tokens or accounts. Thus, the total count of all energy assets of a particular type owned by a client, can be retrieved in a single read operation using Algorithm 2. This algorithm has time and space complexities of O(1) as explained in Section 3.5. In our implementation, the peak throughput for read operation was over 845 TPS with sub second latency as shown in Figure 9.

In the NFT implementation, a client can have several tokens of the same token type. Thus, in order to retrieve a list of all energy assets of a type owned by a client, a bulk read operation needs to be performed using Algorithm 7. As explained in Section 3.5, this algorithm has time and space complexities of O(n) as the time and space required to run this algorithm depends on the number of tokens to be retrieved. In our implementation, we tested the performance for a bulk read operation for 10,000 transactions in each iteration, where each transaction retrieves 10,000 NFT. As shown in Figure 10, the peak throughput was 13 TPS, while average latency was over 18 seconds.

Due to this, while getting all FT for a client can be executed on chain, getting all NFT may need to be executed off-chain. Moreover, Baliga et al. [37] have shown that with the increase in number of total tokens on the state, the read performance suffers marginally, which could be an issue for NFT implementations, for a very large number of tokens in state. Thus, FT have a clear advantage in terms of retrieving all assets of a given type owned by a client.

FT implementations, however, have limitations with concurrent execution of transactions. When two operations try to update the same token, for instance when two buyers try to bid on the same token or when two sellers try to transfer value to the same token, at most one operation will succeed. NFT implementations avoid this problem. When creating or transferring a NFT, a new token is created each time with a distinct key which avoids the problem of contention. Moreover, the issue of accepting multiple bids on the same token does not arise for NFT. Thus, in terms of concurrent execution of transactions, NFT implementations have a clear advantage.

Another implementation specific consideration is the size of the token. As a single FT token accepts multiple bids, the size of the token increases, which can degrade the performance of the network [37,38]. So, specific implementations could consider setting a maximum number of unprocessed bids a token can have at any given time.

Additionally, we have shown that while Create, Bid, Transfer and Redeem operations are similar in performance for FT and NFT, ReCreate and ReTransfer in FT are faster than Create and Transfer respectively, for both FT and NFT. This is due to the fact that the tokens with associated endorsement policies already exist in case of ReCreate and Retransfer operations of FT as discussed in Section 4.2. Thus, neither of the implementations, FT or NFT have a complete advantage over the other in all respects and the choice of implementation will depend on the specific use case.

### 4.4. Limitations and Future Work

In any transaction system, prevention of double spending is an important consideration. Hyperledger Fabric implements Multiversion concurrency control (MVCC) in order to prevent double spending. This means that when multiple operations try to update the same key at the same time, all but one update will fail. FT tokens function like accounts where each client has at most six accounts in our implementation. For instance if two sellers attempt to transfer value to the same token belonging to a buyer, only one of those transfers will complete successfully. The NFT implementation when creating tokens uses the transaction ID and creates new NFT each time with no duplicated keys. Similarly, transfers also create new NFT for the recipient each time, thus preventing contention. Contention is a consideration whenever two processes seek to update a common resource and thus inherent in any account based implementation.

This limitation can be addressed by queuing of transactions. For EUnit and EVUnit, both the buyer and seller are clients of the Transaction Platform. The Transaction Platform application could thus queue the sending of transactions for endorsement and ordering and postpone sending of other transactions that update the same key until the first one has completed or failed. For InUnit, GaUnit, StUnit and EStUnit, the create operation originates from the Power Company and Storage Provider respectively. These could thus be queued by their respective applications.

Alternatively, as tokens are created for each client, for each token type, newer types of tokens or extra clients can be added to create more tokens that can then be simultaneously updated. For tokens that receive a high number of transactions, a running total approach can be considered where several FT of the same token type are created, that are consolidated periodically. The bidding operation on InUnit, GaUnit, StUnit and EStUnit is invoked by clients of the Transaction Platform which can be queued by its application. Similarly, queuing of Transfers can be handled by the Power Company or the Storage Provider as the case may be and queuing of Redeems can be handled by the Transaction Platform.

Thakkar et al. [39] proposed the use of a dependency graph for transactions in order to increase parallel execution in blockchain transactions. Li et al. [40] presented priority based queuing model to reduce the waiting time of blockchain transactions. Investigation of application based contention management through queuing or other methods can be considered for a future work.

## 5. Related Works

Blockchain continues to gain considerable research interest, as the number of publications in the field of blockchain and its applications show a clear upward trend [41]. Features of blockchain such as decentralization, immutability, provenance tracing and self enforcing smart contracts make blockchain suitable to a variety of applications involving information and value exchange, transparency, access control and encoding of business logic for automatic execution. Blockchain was developed as an enabling technology for Bitcoin, a cryptocurrency and thus has several applications in the banking and financial sectors [42]. Blockchain implementations can encompass the business network and provide provenance tracing and can thus facilitate invoice reconciliation and dispute resolution [43]. The immutability of blockchain can increase trust and transparency in transactions and smart contracts can be used to automate transaction flows and processes.

Health care data management is another application area for blockchain. Hasselgren et al. [44] identified access control, data provenance and data integrity as crucial in order to maintain the patient’s privacy during data exchanges between institutions in the healthcare ecosystem. Jiang et al. [45] proposed BlocHIE, a blockchain based platform for privacy and authenticability preserving exchange of medical data. Their solution used two loosely coupled blockchains for storing two different kinds of medical data and proposed two transaction packing algorithms for block creation in order to enhance throughput and fairness. Zhuang et al. [46] proposed the integration of blockchain in order to improve the workflow of health information exchange. Access control was implemented using smart contracts in their solution, which helped them address privacy and data integrity concerns and provide permitted clinicians access to records across multiple medical facilities. Additionally, in light of the global Coronavirus pandemic, blockchain has also been proposed as a solution to address issues such as robust privacy management [47] and provenance based supply chain management [48] for vaccines and contact tracing of affected patients.

Blockchain can also be integrated into industrial internet of things (IIoT) applications due to many of the same reasons that make it a good fit for healthcare and financial domains. Wang et al. [49] analysed the security risks associated with data storage in the IIoT and proposed the use of blockchain in order ensure secure data storage. Wu et al. [50] proposed the integration of blockchain into the supply chain management workflow and implemented a proof of concept on the Hyperledger Fabric for a food traceability system. Chen et al. [51] proposed and implemented a blockchain based access control framework for IoT systems. Jiang et al. [52] proposed the use of blockchain for IIoT data management and presented Fair-Pack, a transaction packing algorithm which succeeded in improving the average response time and fairness of the blockchain as compared to existing algorithms. Bordel et al. [53] presented a theoretical framework to investigate the applicability of blockchain for implementing a privacy and trust preserving solution for storage of data generated by Internet of Things implementations. Dai et al. [54] proposed the integration of blockchain technology to create a privacy preserving platform for Internet of Things. Blockchain mining tasks were delegated to edge nodes through a process of offloading and caching in order to maximize profit and reduce time based on game theory and auction theory. Their approach was shown to perform better than both, a centralized mode as well as a completely decentralized mode where all mining is done by edge nodes, on both profit maximization and time reduction.

Blockchain applications to energy grids have also been extensively studied. Andoni et al. [55] identified that blockchain can add value to the energy grids in the broad areas of billing, sales and marketing, transactions, automation in decentralized applications, smart grid integration, secure information exchange, grid stabilization through usage flexibility, privacy and security, sharing of common resources, competition and transparency. Blockchain is intuitively suited for implementing peer to peer decentralized transaction systems. Li et al. [56] proposed a blockchain based energy trading system that used a credit-based payment scheme for faster transaction confirmation. They presented an optimal pricing strategy based on Stackelberg game for credit-based loans. Additionally, they evaluated their proposal in terms of security and performance to show the efficiency of their solution. Gai et al. [57] proposed a noise-based privacy preserving energy transaction approach by using account mapping to hide user information like location and energy usage. The presented algorithm for noise creation showed an improvement over existing differential privacy approaches in masking for privacy. Aitzhan et al. [58] also focused on implementing transaction security in decentralized smart grid trading. They implemented a token based transaction system to enable users to anonymously negotiate and perform transactions. Paudel et al. [59] proposed a model with competitions between buyers modelled using evolutionary game theory and competition among sellers modelled as a non-cooperative game. The interactions between buyers and sellers were modelled as a M-leader and N-follower Stackelberg game. The evaluation of their approach using simulation showed the convergence of the model and significant financial benefits to the community.

Blockchain has also been used for implementing usage flexibility programs for grid stability. Pop et al. [60] investigated the use of blockchain to store the consumption and production data collected from smart meters. Smart contracts were used to define the usage flexibility expected from each prosumer, with the corresponding incentives and penalties and the rules for maintaining grid stability. Their evaluation of the proof of concept showed that their approach followed the demand signal with high accuracy and reduced the amount of energy flexibility needed. Jindal et al. [61] focused on the security aspects of implementing a demand response mechanism by selecting miner nodes to validate the blocks of energy transactions. The results show that the approach has a low communication and computation overhead. Noor et al. [62] proposed a game theoretic approach to reduce the Peak-to-Average ratio and smooth the load profile. They evaluated their approach using a case study using synthetic data of 15 consumers with multiple appliances and storage capacity. Silvestre et al. [63] proposed using blockchain to record the production and consumption data, calculated the baseline and expected usage flexibility and evaluated their implementation to show the efficacy of their approach.

Tokenization in blockchain is an important topic of investigation as assets on the blockchain are represented in the form of tokens in order to facilitate transactions. Chirtoaca et al. [64] reviewed the applicable metrics for extensibility and reusability of NFT on the Ethereum blockchain and identified the most insightful metrics for these features. Borkowski et al. [65] proposed DeXTT, a protocol for blockchain interoperability for token transactions and showed the logarithmic scalability of their solution with respect to the number of participating nodes. Barreiro-Gomez et al. [66] presented a study of blockchain tokens based on mean-field-type game theory in order to establish a relationship between network characteristics, count of token holders, token price and token supply. Based on their findings, they proposed that the number of tokens in circulation be adjusted in order to capture the risk-awareness and self-regulatory behavior in blockchain economics. Bal et al. [67] proposed NFTTracer, a framework for tracking NFT through modifications. They presented their architecture and algorithms and built a proof of concept but did not present a quantitative analysis of the efficacy of their approach.

Devine et al. [68] proposed a mechanism for renewable energy providers to sell customers future power output in form of NFT on the blockchain. They proposed two ways of structuring these power delivery instruments and evaluated their proposal using a notional market simulation. Regner et al. [69] proposed and described the use of NFT for an event ticketing application. However, a quantitative evaluation of their proposal was not included in their work. Cioara et al. [70] presented an architecture of a blockchain-based smart grid platform and described challenges in implementation of future grid management scenarios such as energy trading, energy flexibility management and virtual power plants. Another work by some of the same authors [30] proposed a blockchain based energy market and described the theoretical framework for implementing the envisioned energy market for NFT assets with features such as registration, bids automation and offers matching.

However, to our knowledge this is the first work that implements a unified energy transaction system in FT as well as NFT versions and draws a comparison between the two implementations in terms of design, algorithmic complexity, limitations and performance.

## 6. Conclusions

In this study we presented a unified blockchain-based system for energy asset transactions among prosumers, EVs, Power Companies and Storage Providers. We implemented and performance tested this system in two versions, one with the energy assets as fungible tokens (FT) and another with non fungible tokens (NFT). We defined operations Create, Bid, Transfer and Redeem for NFT and FT. Additionally, we defined operations ReCreate and ReTransfer for FT, as outlined by the algorithms and token lifecycle presented in Section 3. Based on our analysis, we have the following conclusions:

1. The time and space complexities for Create, Bid, Transfer, Redeem and Read algorithms for NFT are both O(1). The time and space complexities for BulkRead for NFT are both O(n).
2. The time and space complexities for Create, ReCreate, Bid, Redeem and Read algorithms for FT are both O(1), while those for Transfer and ReTransfer algorithms for FT are both O(n).
3. Increasing the permitted queue of unfinished transactions increases the request send rate for all operations. Due to the increase in request send rate, the throughput as well as latency increases for all operations.
4. Increasing the transaction complexity i.e., the number of writes per operation decreases the send rate and throughput achieved for the same queue length and comparable latency.
5. The performance of operations for FT and NFT is similar for Create, Bid, Transfer and Redeem. However, FT operations ReCreate and Retransfer are faster than Create and Transfer for both NFT and FT due to lower number of write operations.
6. For the NFT implementation, Bid and Redeem operations are the fastest, followed by Create and then by Transfer. In the FT implementation, the fastest operations are Bid, Redeem and ReCreate, followed by Create and ReTransfer, followed by Transfer operation.
7. The FT implementation stores the total count of energy assets of a particular type in a single token, while NFT can have multiple tokens with energy assets of the same type. So, performance of retrieving all the energy assets of a particular type was observed to be vastly faster for FT (845 TPS, sub second latency) than for NFT (13 TPS, Latency over 18 s).
8. The NFT implementation avoids contention by creating new tokens with distinct keys whenever Create and Transfer operations are called. Moreover, as two buyers cannot bid on the same token by design, contention is avoided in the NFT implementation. FT tokens function like accounts so, contention is a consideration when two operations try to update the same account.

The implementation and results from the performance testing of the presented energy transaction system with fungible and non fungible tokens provide a proof of concept and show the applicability of blockchain for transacting energy assets in a community based energy infrastructure. Both implementations have comparable performance for all major operations. However, querying for all energy assets owned by a client is a bottleneck for the NFT implementation and could be addressed by moving this operation off-chain. Contention between operations trying to update the same key is a limitation for FT and could be addressed by application based queuing of transactions based on dependency.

No absolute performance related reasons for choosing one implementation over the other were found, and the choice of implementation will thus depend on the specific use case.

## Figures and Tables

**Table 1:** Token issuers, endorsers and usage.

Token Type	Issuer	Endorser	Use
EUnit	Transaction Platform	Storage Provider	Prosumers
EVnit	Transaction Platform	Storage Provider	Prosumer to EVs
InUnit	Power Company	Transaction Platform	Prosumer Incentivization
GaUnit	Power Company	Transaction Platform	Prosumers Gamification
StUnit	Storage Provider	Transaction Platform	Storage token
EStUnit	Storage Provider	Transaction Platform	EV Reward token

**Table 2:** Time and space complexities of algorithms.

Token Type	Operation	Time Complexity	Space Complexity
NFT	Create	O(1)	O(1)
NFT	Bid	O(1)	O(1)
NFT	Transfer	O(1)	O(1)
NFT	Redeem	O(1)	O(1)
NFT	Read	O(1)	O(1)
NFT	BulkRead	O(n)	O(n)
FT	Create	O(1)	O(1)
FT	ReCreate	O(1)	O(1)
FT	Bid	O(1)	O(1)
FT	Transfer	O(n)	O(n)
FT	ReTransfer	O(n)	O(n)
FT	Redeem	O(1)	O(1)
FT	Read	O(1)	O(1)

**Table 3:** Summary of key operations involved in each operation for experimental evaluations.

Token Type	Operation	Write	Set Policy	Read
NFT	Create	1	1	0
NFT	Bid	1	0	1
NFT	Transfer	2	1	1
NFT	Redeem	1	0	1
NFT	Read	0	0	1
NFT	BulkRead	0	0	10,000
FT	Create	1	1	1
FT	ReCreate	1	0	1
FT	Bid	1	0	1
FT	Transfer	2	1	2
FT	ReTransfer	2	0	2
FT	Redeem	1	0	1
FT	Read	0	0	1

## Nomenclature

01. *EUnit*	energy tokens for transacting renewable energy between prosumers
02. *EVUnit*	energy tokens for battery charging transactions between prosumers and electric vehicle owners
03. *InUnit*	reward tokens offered to prosumers by the Power Company for tasks such as consumption estimation
04. *GaUnit*	reward tokens offered to prosumers by the Power Company for energy flexibility in a gamified context
05. *StUnit*	energy storage tokens for managing the community level energy storage transactions
06. *EStUnit*	reward tokens for managing the use of EV batteries as energy storage
07. *NFT Create*	Create operation for non fungible tokens
08. *NFT Bid*	Bid operation used by a buyer to place a bid on a non fungible token
09. *NFT Transfer*	Transfer operation used by seller to transfer value to buyer for non fungible tokens
10. *NFT Redeem*	Redeem operation for non fungible tokens
11. *NFT Read*	Read operation to return a single non fungible token
12. *NFT BulkRead*	Read operation to return multiple non fungible tokens
13. *FT Create*	Create operation involving creating a new fungible token and setting a token level endorsement policy
14. *FT ReCreate*	Create operation involving update to an existing fungible token and no setting of token level endorsement policy
15. *FT Bid*	Bid operation used by a buyer to place a bid on a fungible token.
16. *FT Transfer*	Transfer operation used by seller to transfer value to multiple buyers by creating new fungible tokens
17. *FT ReTransfer*	Transfer operation used by seller to transfer value to multiple buyers by adding value to existing fungible tokens
18. *FT Redeem*	Redeem operation for fungible tokens
19. *FT Read*	Read operation to return a single token for fungible tokens

## Abbreviations

The following abbreviations are used in this manuscript:

FT	Fungible Tokens
NFT	Non Fungible Tokens
EV	Electric Vehicle
VM	Virtual Machine
OSN	Ordering Service Nodes
TPS	Transactions Per Second
QL	Queue Length
SR	Send Rate (TPS)
MVCC	Multi version concurrency control
IIoT	Industrial Internet of Things

## References

[1] Yang X., He L., Xia Y., Chen Y. (2019). Effect of government subsidies on renewable energy investments: The threshold effect. Energy Policy.

[2] Su W., Liu M., Zeng S., Štreimikienė D., Baležentis T., Ališauskaitė-Šeškienė I. (2018). Valuating renewable microgeneration technologies in Lithuanian households: A study on willingness to pay. J. Clean. Prod..

[3] Gautier A., Jacqmin J., Poudou J.C. (2018). The prosumers and the grid. J. Regul. Econ..

[4] Lantero A. US Department of Energy: How Microgrids Work (2014). https://www.energy.gov/articles/how-microgrids-work.

[5] Zhang C., Wu J., Long C., Cheng M. (2017). Review of existing peer-to-peer energy trading projects. Energy Procedia.

[6] Wei Y., Gong Y., Li Q., Song M., Wang X. (2020). Energy Efficient Resource Allocation Approach for Renewable Energy Powered Heterogeneous Cellular Networks. CMC Comput. Mater. Contin..

[7] Zhang C., Wu J., Zhou Y., Cheng M., Long C. (2018). Peer-to-Peer energy trading in a Microgrid. Appl. Energy.

[8] Forbes (2021). The Rising Popularity of Energy Storage as a Service. https://www.forbes.com/sites/pikeresearch/2019/12/06/the-rising-popularity-of-energy-storage-as-a-service/?sh=223abe19a3a7.

[9] Arkhangelski J., Siano P., Mahamadou A.T., Lefebvre G. (2020). Evaluating the economic benefits of a smart-community microgrid with centralized electrical storage and photovoltaic systems. Energies.

[10] Mahmud K., Hossain M.J., Town G.E. (2018). Peak-load reduction by coordinated response of photovoltaics, battery storage, and electric vehicles. IEEE Access.

[11] Uddin M., Romlie M.F., Abdullah M.F., Abd Halim S., Kwang T.C. (2018). A review on peak load shaving strategies. Renew. Sustain. Energy Rev..

[12] Huang W., Zhang N., Kang C., Li M., Huo M. (2019). From demand response to integrated demand response: Review and prospect of research and application. Prot. Control Mod. Power Syst..

[13] Paridari K., Parisio A., Sandberg H., Johansson K.H. Demand response for aggregated residential consumers with energy storage sharing. Proceedings of the 2015 54th IEEE Conference on Decision and Control (CDC).

[14] AlSkaif T., Lampropoulos I., Van Den Broek M., Van Sark W. (2018). Gamification-based framework for engagement of residential customers in energy applications. Energy Res. Soc. Sci..

[15] Nakamoto S. (2008). Bitcoin: A Peer-to-Peer Electronic Cash System. https://bitcoin.org/bitcoin.pdf.

[16] Vangulick D., Cornélusse B., Ernst D. Blockchain for peer-to-peer energy exchanges: Design and recommendations. Proceedings of the 2018 Power Systems Computation Conference (PSCC).

[17] Wang N., Zhou X., Lu X., Guan Z., Wu L., Du X., Guizani M. (2019). When energy trading meets blockchain in electrical power system: The state of the art. Appl. Sci..

[18] Thakur S., Breslin J.G. (2018). Peer to peer energy trade among microgrids using blockchain based distributed coalition formation method. Technol. Econ. Smart Grids Sustain. Energy.

[19] Esmat A., de Vos M., Ghiassi-Farrokhfal Y., Palensky P., Epema D. (2021). A novel decentralized platform for peer-to-peer energy trading market with blockchain technology. Appl. Energy.

[20] Buterin V. (2014). A Next-Generation Smart Contract and Decentralized Application Platform. White Paper.

[21] Androulaki E., Barger A., Bortnikov V., Cachin C., Christidis K., De Caro A., Enyeart D., Ferris C., Laventman G., Manevich Y. Hyperledger fabric: A distributed operating system for permissioned blockchains. In Proceedings of the Thirteenth Eurosys Conference.

[22] Bührer C., Hubli I., Marti E. (2005). The regulatory burden in the Swiss wealth management industry. Financ. Mark. Portf. Manag..

[23] Golang (2021). The Go Programming Language. https://golang.org/.

[24] Gür A.Ö., Öksüzer Ş., Karaarslan E. Blockchain based metering and billing system proposal with privacy protection for the electric network. Proceedings of the 2019 7th International Istanbul Smart Grids and Cities Congress and Fair (ICSG).

[25] Che Z., Wang Y., Zhao J., Qiang Y., Ma Y., Liu J. (2019). A distributed energy trading authentication mechanism based on a consortium blockchain. Energies.

[26] Karandikar N., Chakravorty A., Rong C. Transactive energy on hyperledger fabric. Proceedings of the 2019 Sixth International Conference on Internet of Things: Systems, Management and Security (IOTSMS).

[27] Karandikar N., Chakravorty A., Rong C. RenewLedger: Renewable energy management powered by Hyperledger Fabric. Proceedings of the 2020 IEEE Symposium on Computers and Communications (ISCC).

[28] Statnett (2021). Elcertificates and Guarantees of Origin. https://www.statnett.no/en/for-stakeholders-in-the-power-industry/system-operation/the-power-market/elcertificates-and-guarantees-of-origin/.

[29] Mezquita Y., Gazafroudi A.S., Corchado J., Shafie-Khah M., Laaksonen H., Kamišalić A. Multi-agent architecture for peer-to-peer electricity trading based on blockchain technology. Proceedings of the 2019 XXVII International Conference on Information, Communication and Automation Technologies (ICAT).

[30] Pop C., Antal M., Cioara T., Anghel I. (2020). Trading Energy as a Digital Asset: A Blockchain-Based Energy Market. Cryptocurrencies Blockchain Technol. Appl..

[31] Ausgrid (2021). Community Batteries. https://www.ausgrid.com.au/In-your-community/Community-Batteries.

[32] Thakkar P., Nathan S., Viswanathan B. Performance benchmarking and optimizing hyperledger fabric blockchain platform. Proceedings of the 2018 IEEE 26th International Symposium on Modeling, Analysis, and Simulation of Computer and Telecommunication Systems (MASCOTS).

[33] Ongaro D., Ousterhout J. In search of an understandable consensus algorithm. Proceedings of the 2014 Annual Technical Conference USENIX.

[34] Fabric H. (2021). The Ordering Service. https://hyperledger-fabric.readthedocs.io/en/release-2.3/orderer.

[35] Singh S., Jeong Y.S., Park J.H. (2016). A survey on cloud computing security: Issues, threats, and solutions. J. Netw. Comput. Appl..

[36] Performance H., Group S.W. (2021). Hyperledger Blockchain Performance Metrics White Paper. https://www.hyperledger.org/learn.

[37] Baliga A., Solanki N., Verekar S., Pednekar A., Kamat P., Chatterjee S. Performance characterization of hyperledger fabric. Proceedings of the 2018 Crypto Valley conference on blockchain technology (CVCBT).

[38] Nystrøm F. (2019). Network Performance in Hyperledger Fabric-Investigating the Network Resource Consumption of Transactions in a Distributed Ledger Technology System. Master’s Thesis.

[39] Thakkar P., Nathan S. (2020). Scaling hyperledger fabric using pipelined execution and sparse peers. arXiv.

[40] Li T., Ren Y., Xia J. (2020). Blockchain Queuing Model with Non-Preemptive Limited-Priority. Intell. Autom. Soft Comput..

[41] Zeadally S., Abdo J.B. (2019). Blockchain: Trends and future opportunities. Internet Technol. Lett..

[42] Tapscott A., Tapscott D. (2017). How blockchain is changing finance. Harv. Bus. Rev..

[43] Narayanam K., Goel S., Singh A., Shrinivasan Y., Chakraborty S., Selvam P., Choudhary V., Verma M. Blockchain Based e-Invoicing Platform for Global Trade. Proceedings of the 2020 IEEE International Conference on Blockchain (Blockchain).

[44] Hasselgren A., Kralevska K., Gligoroski D., Pedersen S.A., Faxvaag A. (2020). Blockchain in healthcare and health sciences—A scoping review. Int. J. Med. Inform..

[45] Jiang S., Cao J., Wu H., Yang Y., Ma M., He J. Blochie: A blockchain-based platform for healthcare information exchange. Proceedings of the 2018 IEEE International Conference on Smart Computing (Smartcomp).

[46] Zhuang Y., Sheets L.R., Chen Y.W., Shae Z.Y., Tsai J.J., Shyu C.R. (2020). A patient-centric health information exchange framework using blockchain technology. IEEE J. Biomed. Health Inform..

[47] Ricci L., Maesa D.D.F., Favenza A., Ferro E. (2021). Blockchains for covid-19 contact tracing and vaccine support: A systematic review. IEEE Access.

[48] Antal C.D., Cioara T., Antal M., Anghel I. (2021). Blockchain platform for COVID-19 vaccine supply management. arXiv.

[49] Wang J., Chen W., Wang L., Ren Y., Sherratt R.S. (2020). Blockchain-based data storage mechanism for industrial internet of things. Intell. Autom. Soft Comput..

[50] Wu H., Cao J., Yang Y., Tung C.L., Jiang S., Tang B., Liu Y., Wang X., Deng Y. Data management in supply chain using blockchain: Challenges and a case study. Proceedings of the 2019 28th International Conference on Computer Communication and Networks (ICCCN).

[51] Chen H., Wan W., Xia J., Zhang S., Zhang J., Peng X., Fan X. (2020). Task-Attribute-Based Access Control Scheme for IoT via Blockchain. CMC-Comput. Mater. Contin..

[52] Jiang S., Cao J., Wu H., Yang Y. (2020). Fairness-based Packing of Industrial IoT Data in Permissioned Blockchains. IEEE Trans. Ind. Inform..

[53] Bordel B., Alcarria R., Martin D., Sanchez-Picot A. (2019). Trust provision in the internet of things using transversal blockchain networks. Intell. Autom. Soft Comput..

[54] Dai Y. (2020). Edge computing-based tasks offloading and block caching for mobile blockchain. Comput. Mater. Contin..

[55] Andoni M., Robu V., Flynn D., Abram S., Geach D., Jenkins D., McCallum P., Peacock A. (2019). Blockchain technology in the energy sector: A systematic review of challenges and opportunities. Renew. Sustain. Energy Rev..

[56] Li Z., Kang J., Yu R., Ye D., Deng Q., Zhang Y. (2017). Consortium blockchain for secure energy trading in industrial internet of things. IEEE Trans. Ind. Inform..

[57] Gai K., Wu Y., Zhu L., Qiu M., Shen M. (2019). Privacy-preserving energy trading using consortium blockchain in smart grid. IEEE Trans. Ind. Inform..

[58] Aitzhan N.Z., Svetinovic D. (2016). Security and privacy in decentralized energy trading through multi-signatures, blockchain and anonymous messaging streams. IEEE Trans. Dependable Secur. Comput..

[59] Paudel A., Chaudhari K., Long C., Gooi H.B. (2018). Peer-to-peer energy trading in a prosumer-based community microgrid: A game-theoretic model. IEEE Trans. Ind. Electron..

[60] Pop C., Cioara T., Antal M., Anghel I., Salomie I., Bertoncini M. (2018). Blockchain based decentralized management of demand response programs in smart energy grids. Sensors.

[61] Jindal A., Aujla G.S., Kumar N., Villari M. (2019). GUARDIAN: Blockchain-based secure demand response management in smart grid system. IEEE Trans. Serv. Comput..

[62] Noor S., Yang W., Guo M., van Dam K.H., Wang X. (2018). Energy Demand Side Management within micro-grid networks enhanced by blockchain. Appl. Energy.

[63] Di Silvestre M.L., Gallo P., Sanseverino E.R., Sciumè G., Zizzo G. (2020). Aggregation and remuneration in demand response with a blockchain-based framework. IEEE Trans. Ind. Appl..

[64] Chirtoaca D., Ellul J., Azzopardi G. A framework for creating deployable smart contracts for non-fungible tokens on the Ethereum blockchain. Proceedings of the 2020 IEEE International Conference on Decentralized Applications and Infrastructures (DAPPS).

[65] Borkowski M., Sigwart M., Frauenthaler P., Hukkinen T., Schulte S. (2019). DeXTT: Deterministic cross-blockchain token transfers. IEEE Access.

[66] Barreiro-Gomez J., Tembine H. (2019). Blockchain token economics: A mean-field-type game perspective. IEEE Access.

[67] Bal M., Ner C. (2019). NFTracer: A Non-Fungible token tracking proof-of-concept using Hyperledger Fabric. arXiv.

[68] Devine M.T., Russo M., Cuffe P. (2019). Blockchain Electricity Trading Using Tokenised Power Delivery Contracts. http://aei.pitt.edu/102345/.

[69] Regner F., Urbach N., Schweizer A. (2019). NFTs in Practice–Non-Fungible Tokens as Core Component of a Blockchain-based Event Ticketing Application. https://core.ac.uk/download/pdf/301384284.pdf.

[70] Cioara T., Pop C., Zanc R., Anghel I., Antal M., Salomie I. (2020). Smart Grid Management using Blockchain: Future Scenarios and Challenges. arXiv.

